# 

**Published:** 1901-03-30

**Authors:** 


**Notices of Births, Deaths, and Marriages** are inserted in this column at a charge of 2s. 6d. for an announcement not exceeding four lines.

### MARRIAGE.

On March 20, at St. Andrew's Cathedral, Singapore, by Rev. D. Holland Stubbs. T. Hill Jamieson, M.B.C.M.Edin., of Penang, to Mary, eldest daughter of J. Milnes, Esq., Huddersfield, Yorkshire. (2911)

### NOTICE TO CORRESPONDENTS.

All MSS., Letters, Books for Review, and other matters intended for the Editor should be addressed THE EDITOR, "THE HOSPITAL," THE HOSPITAL BUILDING, 28 & 29 SOUTHAMPTON STREET, STRAND, W.C.

The Editor cannot undertake to return rejected MS., even when accompanied by stamped directed envelope.

### Notice to Advertisers and Subscribers.

All Advertisements, Orders for Copies of THE HOSPITAL, and Business Communications should be addressed to **THE MANAGER (Not to the Editor)**, THE HOSPITAL, 28 & 29 Southampton Street, Strand, London, W.C.

The Cost of Subscription, post free, is as follows:—Per week, 2½d.; per quarter, 2s. 8d.; per half-year, 5s. 3d.; per annum, 10s. 6d. Foreign:—Per annum, 15s. 2d., payable, in all cases, in advance.

**NOTICE.—Vol. XXIX. of "The Hospital" (October 1900, to March, 1901), in handsome cloth binding will shortly be on sale at the Office of this Journal, price, 7s. 6d. Cases for binding the Numbers contained in this Volume 1s. 6d. Index to Vol. XXIX., 2½d., post free.**

**The Monthly Part for April will be ready on April 1st. Price 6d., by post 8d.**

### IMPORTANT NOTICE.

**On account of the Easter Holidays, we beg to announce that we shall go to press with our issue of the 6th APRIL on TUESDAY NIGHT, the 2nd APRIL. Advertisements intended for insertion in this issue should therefore reach us by 4 p.m. ON TUESDAY, the 2nd APRIL.**

### The Student of Medicine.

### APPOINTMENTS.

**John M. Ahern,** L.R.C.P.I., L.R.C.S.I., and L.M., appointed Assistant Medical Officer to the Warneford Asylum, Oxford. **W. T. D. Allen,** M.B., B.Ch.R.U.I., appointed District Medical Officer of the Liverpool Union. **Patrick Callen,** L.R.C.S., L.K.Q.C.P.Irel., appointed Public Vaccinator for Thames, New Zealand. **John F. Christie,** M.B., C.M.Aberd., appointed Medical Officer to the Aberdeen Dispensary. **George Ernest Clemons,** M.D.Edin., appointed Honorary Medical Officer to the Launceston General Hospital, Tasmania. **Martin Dempsey,** M.D., F.R.C.P., appointed Professor of Materia Medica and Therapeutics at the Catholic University School of Medicine, Dublin; also Examiner in Materia Medica at the Royal University, Ireland. **Richard F. Flood,** L.R.C.P.Edin., L.R.C.S.Edin., L.M., &c., appointed House Surgeon to the West Herts Infirmary, Hemel Hempstead. **Arthur George Haydon,** M.B., M.R.C.S., appointed a Civil Surgeon Royal Army Medical Corps; and Medical Officer in charge of the Norfolk Regiment. **L. S. Holmes,** L.R.C.P., L.R.C.S.Edin., appointed Honorary Medical Officer to the Launceston General Hospital, Tasmania. **William A. Kelly,** L.K.Q.C.P.I., L.R.C.S.Irel., appointed Government Medical Officer at Wingham, Western Australia. **Bertram H. Kingsford,** M.B.Lond., M.R.C.S., L.R.C.P., appointed Medical Officer and Public Vaccinator for the Woking District of the Guildford Union, and Certifying Factory Surgeon for the Woking District of Surrey. **G. Liddell,** M.B., M.S. Edin., appointed Public Vaccinator for the District of Otepopo, New Zealand. **E. S. Littlejohn,** M.D.Edin., appointed Visiting Medical Officer to the John Walker Convalescent Hospital, Concord, New South Wales. **S. Macbirnie,** M.B., Ch.M.Glasg., appointed Medical Superintendent to the Yarra Bend Lunatic Asylum, Victoria. **G. F. McCleary,** B.A., M.B., D.P.H.Camb., appointed Medical Officer of Health for the Metropolitan Borough of Battersea. **Allan E. Mahood,** M.B.R.U.I., F.R.C.S.Eng., appointed Surgeon to the Bideford Infirmary. **J. M. Mason,** M.D., D.P.H., appointed Chief Health Officer in New Zealand. **W. P. Morris,** M.D., appointed Assistant Medical Inspector in the Department of Public Health, Victoria. **H. C. Nance,** F.R.C.S.Eng., appointed Surgeon to the Jenny Lind Hospital for Sick Children, Norwich. **J. M'Imery Pardy,** M.B., Ch.B.Melb., appointed Honorary Medical Officer to the Launceston General Hospital, Tasmania. **Antony Roche,** M.R.C.P.I., Professor Catholic University Medical School, appointed Examiner in Medical Jurisprudence and Sanitary Science in the Royal University of Ireland. **F. W. Stansfield,** M.D., Ch.B.Vict., D.P.H.Camb., appointed Public Vaccinator to the Reading Union. **C. E. Sparks,** M.B., appointed District Medical Officer to the Chertsey Union. **W. C. P. Wynne,** L.R.C.P., L.R.C.S.Irel., appointed Certifying Factory Surgeon for the Oxted District of Surrey.

